# Treatment of congenital adrenal hyperplasia in children aged 0–3 years: a retrospective multicenter analysis of salt supplementation, glucocorticoid and mineralocorticoid medication, growth and blood pressure

**DOI:** 10.1530/EJE-21-1085

**Published:** 2022-03-15

**Authors:** Uta Neumann, Annelieke van der Linde, Ruth E Krone, Nils P Krone, Ayla Güven, Tülay Güran, Heba Elsedfy, Sukran Poyrazoglu, Feyza Darendeliler, Tania A S S Bachega, Antonio Balsamo, Sabine E Hannema, Niels Birkebaek, Ana Vieites, Ajay Thankamony, Martine Cools, Tatjana Milenkovic, Walter Bonfig, Eduardo Correa Costa, Navoda Atapattu, Liat de Vries, Guilherme Guaragna-Filho, Marta Korbonits, Klaus Mohnike, Jillian Bryce, S Faisal Ahmed, Bernard Voet, Oliver Blankenstein, Hedi L Claahsen-van der Grinten

**Affiliations:** 1 Institute for Experimental Paediatric Endocrinology, Charité Universitätsmedizin Berlin, Berlin, Germany; 2 Amalia Children’s Hospital, Radboud University Medical Centre, Nijmegen, Netherlands; 3 Amphia Hospital, Breda, The Netherlands; 4 Birmingham Women’s and Children’s Hospital, Birmingham, UK; 5 University of Sheffield, Sheffield Children’s Hospital, Western Bank, Sheffield, UK; 6 University of Health Science Zeynep Kamil Women and Children Hospital, Pediatric Endocrinology, Istanbul, Turkey; 7 Marmara University Istanbul, Istanbul, Turkey; 8 Pediatrics Department, Ain Shams University, Cairo, Egypt; 9 Pediatric Endocrinology Unit, Istanbul Faculty of Medicine, Istanbul, Turkey; 10 Sao Paulo University, Sao Paulo, Brazil; 11 S.Orsola-Malpighi University Hospital, Bologna, Italy; 12 Leiden University Medical Centre, Leiden, Netherlands; 13 Erasmus Medical Centre Rotterdam, Rotterdam, Netherlands; 14 Department of Pediatrics and Steno Diabetes Center Aarhus, Aarhus University Hospital, Aarhus, Denmark; 15 Centro de Investigaciones Endocrinológicas Buenos Aires, Buenos Aires, Argentina; 16 University of Cambridge and Addenbrooke’s Hospital, Cambridge, UK; 17 University Hospital Ghent, Ghent, Belgium; 18 Institute for Mother and Child Healthcare of Serbia ‘Dr Vukan Čupić’, Belgrade, Serbia; 19 Technical University of Munich, Munich, Germany; 20 Klinikum Wels-Grieskirchen, Wels, Austria; 21 Hospital de Clínicas de Porto Alegre, Porto Alegre, Brazil; 22 Lady Ridgeway Hospital, Colombo, Sri Lanka; 23 Institute for Diabetes and Endocrinology, Schneider Children's Medical Center of Israel, Petah-Tikvah, Israel; 24 Sackler School of Medicine, Tel-Aviv University, Tel-Aviv, Israel; 25 Universidade Federal do Rio Grande do Sul, Porto Alegre, Brazil; 26 Queen Mary University of London Barts, London, UK; 27 Otto-von-Guericke Universität Magdeburg, Magdeburg, Germany; 28 University of Glasgow, Glasgow, UK; 29 Voet Consulting, Berlin, Germany

## Abstract

**Objectives:**

International guidelines recommend additional salt supplementation during infancy in classic congenital adrenal hyperplasia (CAH) due to 21-hydroxylase deficiency. The influence of corticoid medication and growth has not been assessed.

**Aim:**

To investigate the current use of salt supplementation, fludrocortisone (FC) and hydrocortisone (HC) dosage as well as weight, height, BMI and blood pressure (BP) in CAH children aged 0–3 years.

**Methods:**

Retrospective multicentre analysis using data from the I-CAH registry. Salt-treated (ST) and non-salt-treated (NST) children were compared regarding FC and HC dosage, weight, height and BP at 0, 3, 6, 9, 12, 18, 24, 30, and 36 months.

**Results:**

We analysed 2483 visits of 331 patients born after year 2000 in 13 countries (male, *n*  = 145) with 203 ST patients (61%). NST children had significantly higher FC dosages at 1.5–4.5 months and higher HC dosages until 1.5 months of age. No differences in weight, length and BP between subgroups were observed. Children of the whole cohort showed increased BMI-SDS during the study period and about half of the reported BP readings were >P95.

**Conclusion:**

In children treated with additional salt supplementation, FC and HC dosages are lower during the first months of life but without differences in weight, length and BP until 3 years of age compared to NST children. All children showed an increase in BMI-SDS and a high rate of BP readings >P95 until 3 years, indicating the start of weight gain and negative effects on blood pressure already in very early life.

## Introduction

Classic congenital adrenal hyperplasia (CAH) is an autosomal recessive disorder, caused by 21-hydroxylase deficiency in 95% of cases, which results in impaired synthesis of glucocorticoids and often mineralocorticoids (1, 2). The severity of the disease depends on the residual enzymatic activity. A complete lack of enzymatic activity causes cortisol and aldosterone deficiency, the salt-wasting (SW) form of CAH. Residual 21-hydroxylase activity of 1–2% generally ensures the production of a sufficient amount of aldosterone to prevent SW crisis and causes classic simple virilizing (SV) CAH (1, 2). In clinical practice, the differentiation between SW and SV CAH can be difficult, especially in the first days of life when hyponatremia is not yet present (3, 4). Even, if the genotype is known, a wide phenotypic variability can be seen (3, 4). In countries where classic CAH is detected within a neonatal screening programme, treatment starts with hydrocortisone (HC) and fludrocortisone (FC) before clinically relevant SW occurs (1). To compensate for additional SW from immature renal tubules, international guidelines recommend the use of additional salt supplementation of about 2 mmol/kg/day in infancy under close monitoring of blood pressure, serum sodium, potassium and plasma renin activity (PRA) (1, 5). Nevertheless, supplementation of sodium chloride is not routinely given in all expert centres (6).

In rare, inherited diseases such as CAH with a prevalence of 1:14 000–1:18 000, the use of international registries offers a good opportunity to investigate a significant number of affected patients (7). With the use of the International CAH (I-CAH) registry, the current use of additional salt supplementation in a big international cohort of young children aged 0–3 years was examined. Furthermore, we compared salt-treated (ST) and non-salt-treated (NST) children according to FC and HC dosage, height, weight and BP to gain further insights into the still controversial discussion on additional salt therapy in CAH during infancy.

## Patients and methods

### Patient selection

We performed a retrospective, multicentre study by using data from the I-CAH registry (https://home.i-cah.org/), an international database of pseudonymized information on patients with CAH, which is approved by the National Research Ethics Service in the United Kingdom as a research database of information that is collected as part of routine clinical care (6). The data within this registry are deposited by clinicians following informed consent from patients or guardians. When the study was performed, the registry contained data from 1072 patients with CAH with a median age of 16 years from 70 centres from 27 countries in 5 continents. Quality assessment of the registry showed a high degree of validity, consistency and accuracy (8).

The current study analysed data of children born after the year 2000 with CAH due to 21-hydroxylase deficiency and treated with HC and FC. No pre-specified visit schedule was used and the data were organized and analysed based on patients’ collected age. Data used for statistical analyses from the basic module were birth weight, birth length and mutation analysis. Data used from the longitudinal module of the first 3 years of life were height, weight, dosage of medication (FC, HC and salt supplementation) and BP at different time points.

### Data analysis

Automated data cleaning reassures that sites could only enter values in specific ranges. After data extraction and before any statistical analysis, the data were checked for accuracy and plausibility. In case of inaccuracy or incomplete data files, the centres were asked to review and complete the dataset. Missing data were not replaced. Only patients treated with FC for at least 3 months were included in the study. Visits at different time points were summarized in age ranges: birth (0–<1.5 months); 3 months (1.5–<4.5 months); 6 months (4.5–<7.5 months); 9 months (7.5–<10.5 months); 12 months (10.5–<15 months); 18 months (15–<21 months); 24 months (21–<27); 30 months (27–<33); 36 months (33–42 months). If more than one measurement was collected within a single age range, the average of these measurements was used for analysis.

For comparative analysis, patients were divided into two groups: ST and NST. Salt treatment was defined as additional salt supplementation given at least between two visits between birth and 365 days of life.

Equivalent dose (ED) for glucocorticoid and mineralocorticoid action was calculated by using the formulas: ED-HC (glucocorticoid equivalent dose) = HC (mg) + FC (µg) × 0.0125 and ED-FC (mineralocorticoid equivalent dose) = FC (µg) + HC (mg) × 5, assuming a glucocorticoid potency of FC of 12.5 and a mineralocorticoid potency of HC of 1/200 (9).

Parental target height was calculated according to the formula of Hermanussen (10). Systolic and diastolic BP were recalculated into percentiles for age, height and sex (11). Z-scores for weight, height, BMI were calculated using the World Health Organization (WHO) percentiles (https://www.who.int/childgrowth/standards/height_for_age/en/), which were released in 2006. These includes percentiles and z-score curves for boys and girls aged 0–60 months to assess the weight-for-age, length/height-for-age, weight-for-length/height (45–110 cm and 65–120 cm, respectively) and BMI-for-age (12, 13). The weight-for-length and -height and BMI-for-age yield very similar results, which suggest comparability between indicators for assessing overweight and obesity in preschool-age children (14). Dosage of HC was calculated as mg/m^2^/day and FC was calculated as µg/day. BMI was calculated as weight in kilograms divided by the square of height in metres (kg/m^2^). The classification according to the information on *CYP21A2*-gene mutation analysis given in the I-CAH registry was divided into SW CAH (groups 0 or A), simple virilizing CAH (group B) or non-classic CAH (group C). For this categorization, several references on genotype–phenotype correlation based on* in vitro CYP21A2* activity correlation were used (3, 15, 16, 17).

### Statistical analysis

At first, a whole group analysis was done including all children with FC-treated CAH, independent of salt supplementation in the first year of life. In a second step, the two subgroups, ST and NST, were compared with regard to weight, height, BMI, HC and FC dosage and BP at the defined time points during the first 3 years of life. If no information on additional salt supplementation was available, patients were excluded from the two-group analysis but were included in whole group analysis. To investigate the influence of HC and FC dosages given in the first 1.5 months of life, a pairwise correlation analysis of that dosage and weight, height, BMI and BP at 1, 2 and 3 years of life was performed.

All computations were performed using the statistical software SAS, Version 9. The collected data were presented as sample size, mean ± s.d. Categorical variables were represented by absolute and relative frequencies and were compared through Fisher’s exact test. Continuous variables were compared between the NST and ST groups using the Student’s *t* test, and Pearson’s correlation coefficients were used to assess the relationships between outcome variables. No non-parametric test was performed, all variables were regarded as being normally distributed. All statistical tests were two-tailed, and significance was set at the 0.01 level. *P*-values below 0.01 could be seen as an indicator for possible group differences.

## Results

A total of 2483 visits of 331 patients (145 males) with classic CAH due to 21-hydroxylase deficiency born after the year 2000 and treated in 22 centres from 13 countries were analysed. Results of *CYP21A2*gene analysis in 217 patients were given, among these were not classifiable in 14 patients. There were no differences in the frequency of genetic groups between the ST and NST group: genetic group 0 or A (ST *n*  = 82; NST *n*  = 90), B (ST *n*  = 11, NST *n*  = 18) and C (ST *n*  = 5, NST *n*  = 7).

### Salt supplementation

**Figure 1:**
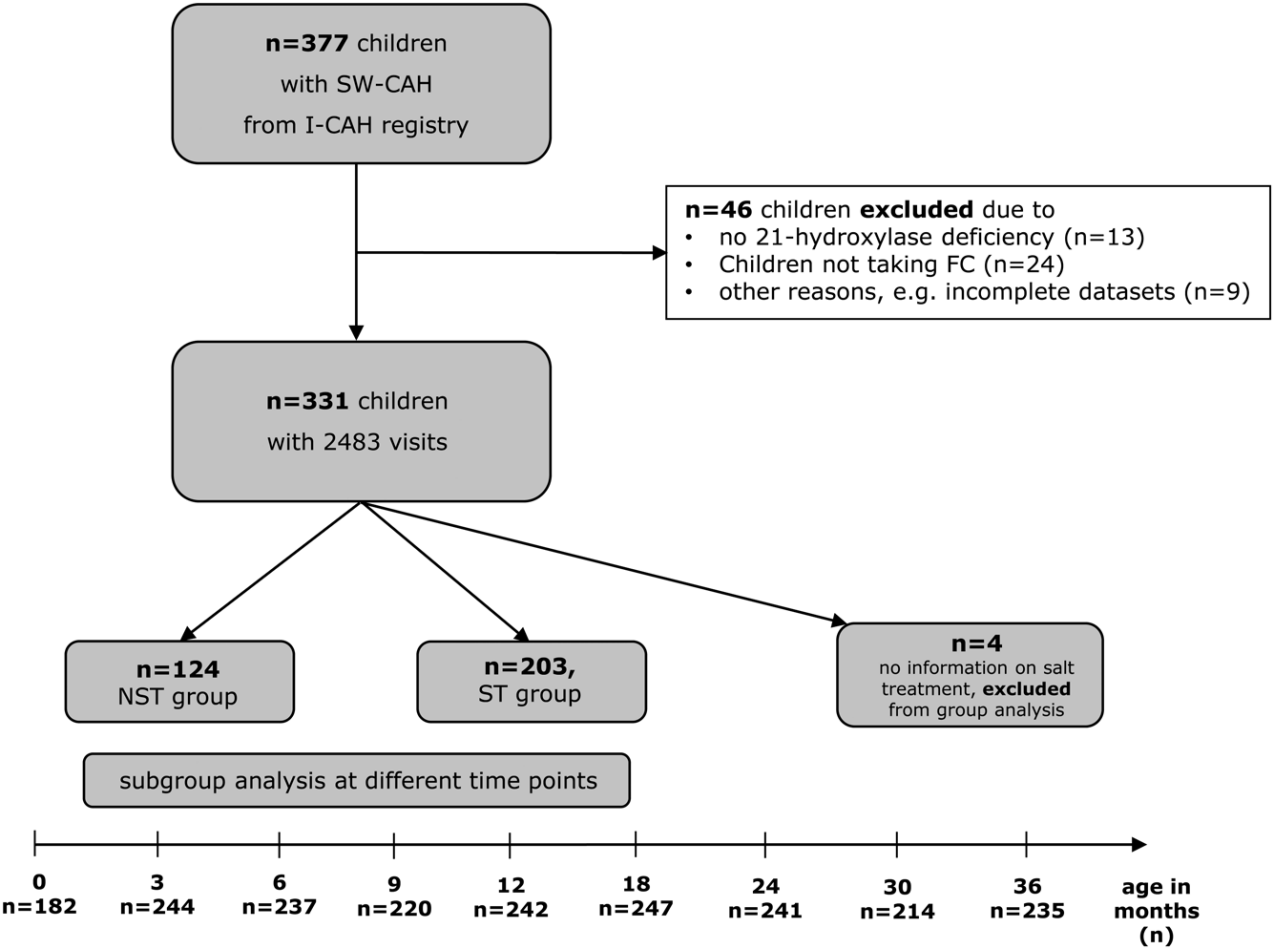
Flowchart on data processing and number of patients. Data extraction revealed 1072 patients with CAH but only 377 children younger than 3 years of age suffering from SW-CAH. SW-CAH, salt wasting CAH; I-CAH registry, International congenital adrenal hyperplasia registry; FC, fludrocortisone; ST, salt treated at least between two time points between birth and 365 days of life; NST, non-salt-treated.

In the whole cohort, salt supplementation was given to 203 patients (61%, ST), and 124 patients were not salt supplemented (38%, NST). In four patients, information on salt treatment was not available (1%) (Fig. 1).

In 92% of ST children, salt supplementation started within the first 6 weeks of life, and in 15% of ST-children, salt supplementation remained after 2 years of age. The mean dose of salt documented in 151 children at different time points lay between 0.81 and 1.03 g/day (3.3–4.7 mmol/kg/day).

### FC dosing

In the whole cohort, FC dosage was highest in the first 4.5 months of age (114.3 ± 61.0 µg/day, *n*  =228) with a continuous decline in dosage until 3 years of age: dosage at 12 months: 99.5 ± 45.4 µg/day, *n*  = 234; at 24 months: 94.4 ± 54.1 µg/day, *n*  = 238; at 36 months: 85.8 ± 40.9 µg/day, *n*  = 229). Comparison between the two treatment groups revealed a significantly higher dosage of FC in the NST group compared to the ST group between 1.5 and 4.5 months of age (ST, 103.9 ± 61.3 µg/day, *n*  = 142; NST, 131.3 ± 56.8 µg/day, *n*  = 86, *P*  < 0.001). From 2 to 3 years of age, the FC dosage was significantly higher in the ST group compared to NST group (Table 1).

**Table 1:** Daily hydrocortisone (HC) dose, fludrocortisone (FC) dose, equivalent glucocorticoid action of HC and FC (ED HC) and equivalent mineralocorticoid action of FC and HC (mean ± s.d., (*n*)).

Age/medication	NST	ST	Total	*P*-value*
0 month				
HC (mg/m^2^)	28.5 ± 9.1 (39)	21.2 ± 13.6 (80)	23.6 ± 12.8 (119)	<0.001
ED HC (mg/m^2^)	34.7 ± 10.1 (39)	25.5 ± 11.5 (76)	28.6 ± 11.8 (115)	<0.001
FC (µg/day)	111.5 ± 41.7 (50)	96.4 ± 54.6 (120)	100.8 ± 51.5 (170)	0.08
ED FC (µg/day)	145.2 ± 41.2 (48)	111.6 ± 51.6 (105)	122.1 ± 50.7 (154)	<0.001
3 months				
HC (mg/m^2^)	17.3 ± 7.0 (77)	17.1 ± 8.8 (114)	17.2 ± 8.0 (194)	0.89
ED HC (mg/m^2^)	22.6 ± 7.8 (77)	20.9 ± 7.9 (111)	21.6 ± 7.9 (191)	0.10
FC (µg/day)	131.3 ± 56.8 (86)	103.9 ± 61.3 (142)	114.3 ± 61.0 (228)	<0.001
ED FC (µg/day)	157.9 ± 59.4 (83)	124.6 ± 58.1 (119)	138.3 ± 60.4 (205)	<0.001
6 months				
HC (mg/m^2^)	14.4 ± 6.1 (81)	13.8 ± 5.3 (109)	14.0 ± 5.7 (192)	0.44
ED HC (mg/m^2^)	18.3 ± 6.3 (79)	17.3 ± 5.8 (109)	17.6 ± 6.0 (190)	0.27
FC (µg/day)	115.8 ± 47.1 (86)	103.7 ± 65.9 (144)	108.2 ± 59.7 (230)	0.14
ED FC (µg/day)	141.9 ± 47.6 (81)	123.3 ± 63.9 (118)	130.8 ± 58.2 (201)	0.06
9 months				
HC (mg/m^2^)	12.4 ± 4.4 (77)	12.8 ± 4.1 (97)	12.7 ± 4.5 (175)	0.52
ED HC (mg/m^2^)	15.6 ± 4.4 (76)	15.9 ± 4.7 (96)	15.9 ± 4.9 (173)	0.71
FC (µg/day)	106.7 ± 45.1 (82)	102.0 ± 48.7 (129)	103.8 ± 47.3 (211)	0.48
ED FC (µg/day)	130.7 ± 44.0 (79)	124.9 ± 47.5 (101)	127.9 ± 46.3 (181)	0.55
12 months				
HC (mg/m^2^)	12.0 ± 3.1 (88)	11.9 ± 4.1 (107)	12.1 ± 4.0 (198)	0.86
ED HC (mg/m^2^)	14.8 ± 3.2 (87)	14.7 ± 4.5 (107)	14.9 ± 4.3 (197)	0.54
FC (µg/day)	98.7 ± 37.1 (92)	100.1 ± 50.1 (142)	99.5 ± 45.4 (234)	0.82
ED FC (µg/day)	124.5 ± 35.9 (89)	124.0 ± 44.2 (115)	124.7 ±40.9 (207)	0.85
18 months				
HC (mg/m^2^)	11.3 ± 2.7 (85)	12.0 ± 4.1 (119)	11.8 ± 4.0 (207)	0.14
ED HC (mg/m^2^)	13.6 ± 2.9 (84)	14.5 ± 4.4 (119)	14.2 ± 3.9 (206)	0.51
FC (µg/day)	92.5 ± 40.2 (92)	97.0 ± 48.7 (151)	95.3 ± 45.7 (243)	0.45
ED FC (µg/day)	117.6 ±36.3 (87)	122.2 ± 44.1 (123)	120.8 ± 41.1 (213)	0.26
24 months				
HC (mg/m^2^)	11.3 ± 2.5 (80)	11.5 ± 4.2 (110)	11.5 ± 3.6 (192)	0.74
ED HC (mg/m^2^)	13.3 ± 2.7 (80)	13.8 ± 4.4 (110)	13.6 ± 3.8 (192)	0.59
FC (µg/day)	82.9 ± 37.1 (93)	101.8 ± 61.6 (145)	94.4 ± 54.1 (238)	<0.01
ED FC (µg/day)	114.0 ± 37.5 (87)	127.2 ± 48.9 (113)	121.4 ± 44.3 (202)	0.03
30 months				
HC (mg/m^2^)	11.1 ± 2.6 (81)	11.1 ± 3.2 (102)	11.1 ± 3.0 (186)	0.94
ED HC (mg/m^2^)	12.8 ± 2.7 (79)	13.2 ± 3.3 (101)	13.1 ± 3.1 (183)	0.42
FC (µg/day)	79.3 ± 30.9 (84)	94.3 ± 45.7 (123)	88.2 ± 41.0 (207)	<0.01
ED FC (µg/day)	112.1 ± 33.2 (81)	124.9 ± 46.7 (102)	119.2 ± 41.5 (186)	0.02
36 months				
HC (mg/m^2^)	11.0 ± 2.6 (89)	11.8 ± 3.8 (102)	11.5 ± 3.4 (194)	0.13
ED HC (mg/m^2^)	12.6 ± 2.7 (89)	13.7 ± 3.9 (101)	13.3 ± 3.5 (193)	0.16
FC (µg/day)	76.9 ± 32.8 (96)	92.3 ± 44.9 (133)	85.8 ± 40.9 (229)	<0.01
ED FC (µg/day)	110.4 ± 33.2 (90)	130.0 ± 46.3 (105)	121.5 ± 41.8 (198)	<0.001

*P-value: group difference tested between NST and ST group. Significance was set at the 0.01 level (bold values).

ED HD, glucocorticoid equivalent dose of HC and FC; ED FC, mineralocorticoid equivalent dose of HC and FC; FC, fludrocortisone dosage; HC, hydrocortisone dosage; NST, children did not receive salt supplementation at any time point between birth and 365 days of life; ST, children treated with salt at least between two time points between birth and 365 days of life.

### HC dosing

All children in analysis received HC with a mean dose of above 15 mg/m^2^/day in the first 4.5 months of life. From 6 months until 3 years of age, HC dosage was within the recommended dose range of 10–15 mg/m^2^/day with a decline until 3 years of age (11.5 ± 3.4 mg/m^2^, *n*  = 194) (Table 1). Comparison of the ST and NST group revealed a significantly higher dosage of HC in the NST group only until 1.5 months of life (ST, 21.2 ± 13.6 mg/m^2^, *n*  = 80; NST, 28.5 ± 9.1 mg/m^2^, *n*  =39, *P*  < 0.001) (Table 1).

### Growth

#### Body weight/BMI

**Figure 2:**
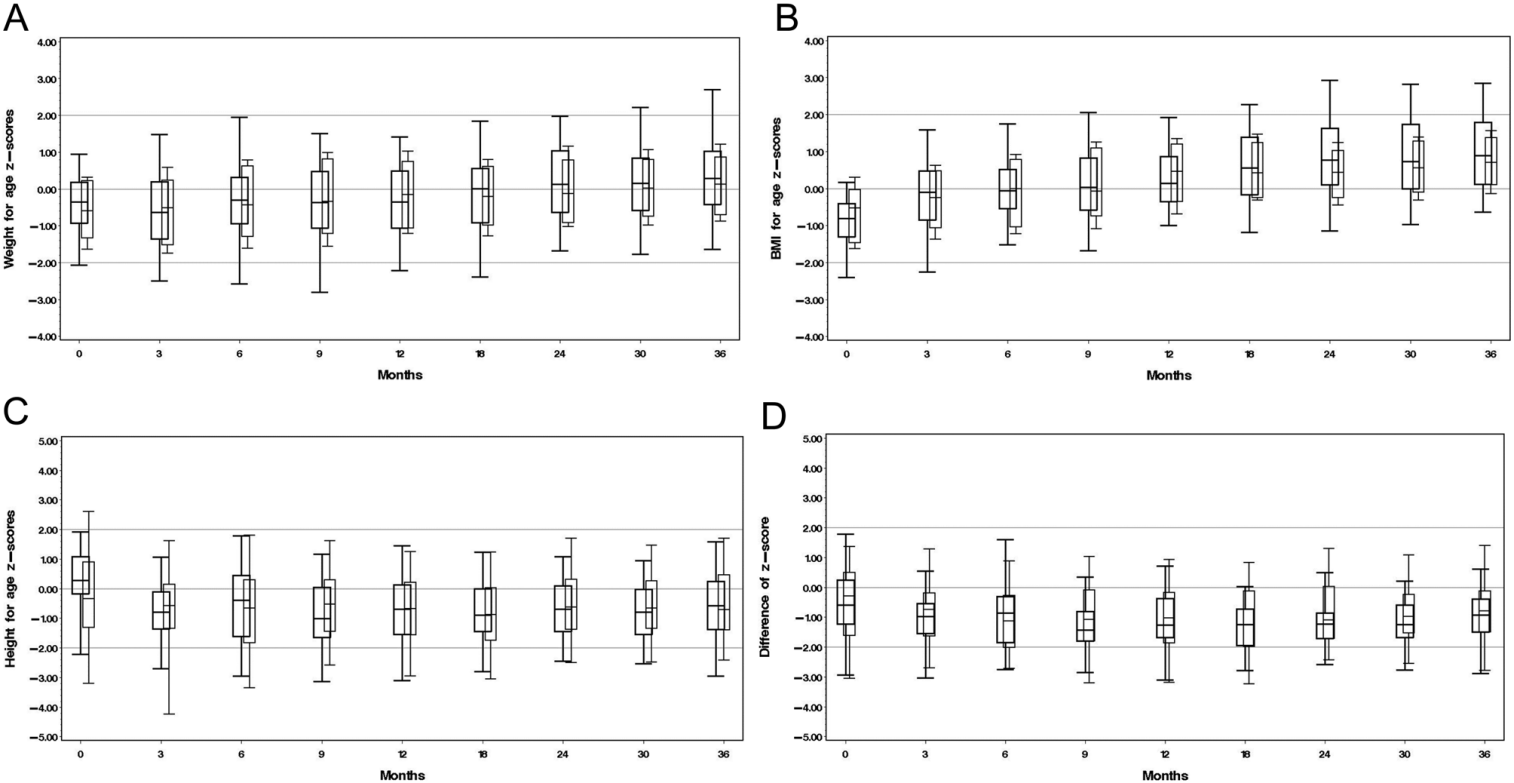
Box plots of weight for age z-scores (A), BMI for age z-scores (B), height for age z-scores (C) and difference between height for age z-scores and parental target height z-scores (D) in ST and NST group from birth to 3 years of life. The bottom and top edges of the box are located at the sample 25th and 75th percentiles. The length of the whiskers is the 5th percentile low and 95th high. ST, children treated with salt at least between two time points between birth and 365 days of life (right-sided boxes); NST, children did not receive salt supplementation at any time point between birth and 365 days of life (left-sided boxes, bold).

In the total cohort, we found an increase in mean weight-for-age-z-score from −0.61 SDS at birth to 0.26 SDS at 3 years of age. Similarly, an increase in BMI-for-age-z-score of 1.6 s.d. during the first 3 years of life was shown in the whole study group (mean BMI at birth: −0.71 SDS, mean BMI at 3 years: 0.86 SDS). There was no difference in birth weight between the two subgroups (ST, 3323.0 ± 677.4 g, *n*  =183; NST, 3379.6 ± 542.6 g, *n*  = 119, *P* = 0.44) and in weight or BMI until the age of 3 years (Fig. 2A and B).

#### Height

In the total cohort, height z-scores decreased during the first months of life with a minimum of −0.86 SDS at 1.5 years of life. There was no difference in birth length between the two subgroups (ST, 50.2 ± 3.5 cm, *n*  =98; NST, 50.7 cm ± 3.0 cm, *n*  =94, *P* = 0.29). After the initial decline in height z-scores, all patients showed a stable height z-score without any group differences (Fig. 2C). Compared to mean parental target height, height z-scores decreased slightly but were within the parental target range until 3 years of age without any difference between the groups (Fig. 2D).

#### Blood pressure

In 2483 visits, a total of 698 systolic BP measurements (28.1%) of 221 children and 684 diastolic BP measurements (27.5%) of 219 children were analysed. In 221 children, there was at least 1 BP measurement, and in 149 children, there were at least 2 BP measurements, and in 110 children, there were at least 3 BP measurements analysed.

At 18 months of age, more than half of all children showed a systolic BP > P95 (57%, *n*  = 38) and a diastolic blood pressure > P95 in 75%, (*n* = 50). In half of all children with listed blood pressure (52%), a diastolic BP > P95 was still detected at the age of 2.5 years (*n* = 42) (Supplementary Table 1 and Supplementary material, see section on supplementary materials given at the end of this article). There were no differences in systolic or diastolic BP at any time point between the ST and NST groups (Supplementary Table 2 and Supplementary material).

### Effect of HC and FC dosage on weight, height, BMI and BP

Pairwise correlation analysis of HC and FC dosage given at birth and weight, height, BMI and BP at 1, 2 and 3 years of life revealed a significant positive correlation of total HC dosage (mg/m^2^) (r = 0.44, *P*  < 0.001), FC dosage (r = 0.41, *P* = 0.002), total mineralocorticoid action (ED FC) of FC and HC (r = 0.50, *P*  < 0.001) as well as total glucocorticoid action (ED HC) of FC and HC (r = 0.47, *P* = 0.004) with systolic BP at 1 year of age. The diastolic BP at 1 year of age was positively correlated with total mineralocorticoid action of HC and FC dosage (ED FC) (r = 0.30, *P* = 0.04).

## Discussion

This is the largest multicentre study so far describing the common practice of additional salt supplementation as well as HC and FC treatment and height, weight, BMI and BP in children with FC-treated CAH during the first 3 years of life. Children with additional salt supplementation had lower HC dosages in the neonatal period and lower FC dosages between 1.5 and 4.5 months of age. However, no differences between subgroups could be detected regarding weight, height and BP in the first 3 years of life. We observed HC dosages exceeding 17 mg/m^2^/day in the first months of life in the total cohort. Furthermore, in the first 2 years, nearly half of the BP measurements in the total cohort were elevated.

Current international guidelines recommend additional salt supplementation in classic CAH during infancy (1). However, the influence of corticoid medication and growth has not been assessed so far. The neonatal kidney is characterized by a lower glomerular filtration rate and immature tubules compared to the adult kidney (18). Furthermore, there is a physiologic transient and partial aldosterone resistance in healthy neonates accompanied with slightly decreased sodium levels, slightly elevated potassium levels, high aldosterone and renin levels and a high urinary sodium loss (19). In addition, elevated 17-hydroxy-progesterone (17OHP) levels that function as biomarker of the disease have an antagonistic effect on the mineralocorticoid receptor. All these factors may contribute to the increased risk of SW explaining the need for higher FC dosages in early infancy (20). Furthermore, it is known that human milk contains only low salt concentrations that are probably not sufficient to compensate for potential salt loss in aldosterone deficiency (21).

The current guidelines recommend an FC dosage of 0.05–0.2 mg/day throughout life without a clear specification for the young age group, but the therapy must be reassessed periodically based on blood pressure, serum sodium, potassium and plasma renin activity (PRA) (1). The sensitivity of the mineralocorticoid receptor to mineralocorticoids increases quickly within the first months of life. In our cohort, FC dosages decreased within the first 3 years of life from a mean dose of 100 µg per day in the first weeks of life to a median dose of 85 mcg after 3 years, most decreasing in the NST group. The difference in FC dosage between the subgroups, which was found after 2 years of age, could not be explained by our data. In our analysis, only children treated with FC were included and the distribution of mutations of group 0 and A was equal in both groups. From phenotype–genotype studies, it is known that certain mutations cause different CAH phenotypes (3). This could be one possible explanation of treatment with FC and salt supplementation in children with a mutation not classified as a 0 or A mutation.

The need for FC treatment depends on the residual enzymatic activity and is age-dependent. Frequent measurements of different clinical parameters like renin or plasma renin activity (PRA), serum sodium and potassium concentration and BP are recommended (1, 22). In the study of Bonfig* et al*., more than 50% of children with CAH show a hypertensive systolic BP at the age of 1.5 years (23) and a higher risk of developing hypertension in older children is reported especially when treated with FC (23, 24, 25). Similarly, in our cohort, increased rates of hypertensive BP readings in nearly 50% of children within the first 1.5 years of life were shown. However, unfortunately, only in 25% of visits, BP values were available for analysis. In one study of 24 children under 1 year of age, no significant increase in BP was found, but the low number of patients was discussed as possible reason (26). In our study, the systolic BP at 1 year of age was positively correlated to the total glucocorticoid and mineralocorticoid action of HC and FC dosages in the first 1.5 months of life. An inappropriate use of mineralocorticoids and salt may be a risk factor for hypertension already in early infancy (23, 26, 27, 28, 29). A meta-analysis revealed a reduction of BP in normotensive and hypertensive non-CAH individuals after reducing salt intake (30). This effect was also shown in infants after a low sodium diet during the first 6 months of life, leading to a reduced BP in infants compared to children with a normal sodium intake. The group differences in BP remained after 15 years of follow-up (31). In contrary, increased BP levels in early life were predictive of adult hypertension (32).

In our analysis, 90% of ST children received additional salt during the first 6 months of age. At 1 year of age, still, 51% of ST patients were treated with additional salt, and in 15% of children of the ST group, the salt supplementation continued after 2 years of age. The rationale for long-term salt supplementation is unclear, as a sufficient salt supplementation is completely covered by normal feeding patterns in Western countries starting at the end of the first year (33). In general, salt supplementation in the first weeks of life may have positive effects on weight gain and growth, while salt loss in humans causes growth failure (34). Especially in preterm infants with physiological salt loss, the neurodevelopmental outcome was better in salt-supplemented children (35).

We observed a HC dose above 15mg/m^2^/day in the total study group during the first 4.5 months of life, and the NST group was treated with nearly twice the recommended HC dose (mean 28 mg/m^2^) in the first 1.5 months of age, which was significantly higher compared to ST group. Bonfig* et al*. also described higher, although not significant, HC dosages in the NST group during the first 3 months of life in CAH infants (6). In clinical practice, stress doses of HC are often used at the beginning of treatment in attempt to quickly normalize the elevated 17-OHP and androstenedione levels probably explaining the higher dosages of HC in the first few months of life. One further possible reason for the observed high HC dosages in all children until 4.5 months of age is the limited availability of preparations for young children in adequate doses. HC was only available in 10 mg tablets in Europe and 5 mg tablets in the United States for a long time. Meanwhile, licenced HC preparations are available to treat children with CAH from birth with dosage as low as 0.5 mg (36). This is important as high dosage of HC is a known risk factor for decreased growth velocity (37, 38). In one study, CAH children treated with a HC dose of 10 mg/m^2^ grow within the parental target height range during the first 3 years of life (39). In our study, children showed a decrease in mean height z-score and an increased difference between height parental target height z-score during the first 6–9 months of age. After the first year of life, stable growth and height z-score were seen. Therefore, high dosages of FC and HC in the beginning might be possible risk factors for the decline in height z-score during the first year of life with possible long-term consequences on final height.

Additionally, all children showed an increase in BMI within the first 3 years of age in our study. This could be enhanced by supraphysiological dosages of glucocorticoids, which could increase the CV risk profile in CAH patients already at a young age (40). Next to pharmacotherapy, the increase in BMI is also an independent risk factor for increased BP in childhood (11). Further comparative studies are necessary to investigate the long-term effect of CAH therapy in childhood on BP, weight, metabolic parameters and body composition in adults with CAH.

There are several limitations in our study. We used retrospective data from the I-CAH registry entered by different centres with the risk of deficient and incomplete data entry. The quality of the I-CAH registry was assessed against recent standards set for registries for rare conditions and revealed a high level of compliance with these standards (8). Although this is the largest multicentre study on the use of additional salt in young children with CAH, at some visits, only a small number of data, for example, BP readings, are available. Additionally, measurement of BP during early infancy is challenging and high readings might be due to artefacts. In the I-CAH registry, there is no information given on the procedure of blood pressure measurements, for example, multiple measurements or feed status. Alternatives in measurement of BP are needed to get reliable results. At least, an ambulatory BP monitoring carried out over a 24-h period should confirm the suspicion of hypertension as recommended in the guidelines for the management of hypertension in childhood as soon as possible (41). Until this time, regular BP measurements during frequent visits in the outpatient clinic and the average of three measurements at one time should be taken. To evaluate the influence of FC on BP, the analysis of renin or PRA may be useful. This might give further insights into therapy adaptation of FC treatment throughout the first years of life. Unfortunately, this analysis could not be done due to few laboratory data in the I-CAH registry. The incidence of an adrenal crisis was also not examined in the current study.

In our study, we described the current practice of therapy with glucocorticoids, mineralocorticoids and salt supplementation in CAH children aged 0–3 years. With the results, we aim to sensitize clinicians to scrutinize the therapy at every visit of a growing child.

In conclusion, in CAH children treated with additional salt, the FC and HC dosages were lower during the first 4.5 months of life without differences in weight, length and BP until 3 years of age compared to NST children. Therefore, additional salt supplementation during the first 3–6 months of life might be effective to reduce glucocorticoid and mineralocorticoid dosages. Prospective studies are necessary to confirm this hypothesis. Children of the whole cohort showed an increase in weight and a high rate of BP > P95 until 3 years, indicating that weight gain and negative effects on blood pressure due to glucocorticoid and mineralocorticoid therapy may start already in early life.

## Supplementary Material

Supplementary Table 1: Percentage of Blood pressure (BP) >95% percentile (n) at different time points from birth to 3 years of age compared by salt replacement status given in % (n).

Supplementary Table 2: Blood pressure (RR) percentiles from birth to 3 years of age compared by salt replacement status (mean ± SD (n)).

## Declaration of interest

Hedi L Claahsen-van der Grinten is on the editorial board of EJE. Hedi L Claahsen-van der Grinten was not involved in the review or editorial process for this paper, on which he/she is listed as an author. The other authors declare that there is no conflict of interest.

## Funding

This project has received support from the I-CAH Registry project that has received unrestricted education grants from Diurnal Ltd and Neurocrine Biosciences http://dx.doi.org/10.13039/100014593. The initial development of the Registry was supported by the Medical Research Council http://dx.doi.org/10.13039/501100000265 (G1100236), the Seventh European Union Framework Program (201444) and the European Society for Paediatric Endocrinology http://dx.doi.org/10.13039/100010381 Research Unit.

## Author contribution statement

O Blankenstein and H L Claahsen-van der Grinten: shared senior authorship.

## Data availability

The datasets generated or analysed during the current study are not available publicly but available to access through a data sharing agreement available at https://idsdorg.files.wordpress.com/2020/05/i-dsd-i-cah-data-sharing-agreement.docx.
