# Supplementary material for: Treatment of congenital adrenal hyperplasia in children aged 0–3 years: a retrospective multicenter analysis of salt supplementation, glucocorticoid and mineralocorticoid medication, growth and blood pressure
Source: Eur J Endocrinol. 2022 Mar 15;186(5):587–96. doi: 10.1530/EJE-21-1085 (PMC9066592; doi:10.1530/EJE-21-1085)
Supplement: Supplementary Table 1: Percentage of Blood pressure (BP) >95% percentile (n) at different time points from birth to 3 years of age compared by salt replacement status given in % (n). [file supplementary_table_1.pdf]

Supplementary Table 1: Percentage of Blood pressure (BP) >95% percentile (n) at different time points from birth to 3 years of age compared by salt replacement status given in % (n).

Age in months		total	NST	ST	p-value*
0	Systolic RR	44% (10)	40% (2)	44% (8)	1.00
	Diastolic RR	50% (11)	80% (4)	41% (7)	0.31
3	Systolic RR	54% (32)	57% (8)	53% (24)	1.00
	Diastolic RR	60% (35)	64% (9)	59% (26)	1.00
6	Systolic RR	52% (34)	68% (13)	45% (21)	0.11
	Diastolic RR	73% (47)	89% (17)	67% (30)	0.07
9	Systolic RR	46% (30)	59% (10)	42% (20)	0.27
	Diastolic RR	59% (38)	76% (13)	53% (25)	0.15
12	Systolic RR	48% (34)	58% (14)	43% (20)	0.22
	Diastolic RR	66% (46)	67% (16)	65% (30)	1.00
18	Systolic RR	57% (38)	65% (13)	53% (25)	0.43
	Diastolic RR	75% (50)	70% (14)	77% (36)	0.56
24	Systolic RR	39% (35)	46% (13)	36% (22)	0.36
	Diastolic RR	58% (50)	54% (15)	59% (35)	0.65
30	Systolic RR	35% (28)	39% (12)	35% (16)	0.63
	Diastolic RR	52% (42)	61% (19)	45% (23)	0.18
36	Systolic RR	19% (22)	24% (12)	15% (10)	0.34
	Diastolic RR	33% (37)	33% (16)	32% (21)	1.00

*p-value: group difference tested between children not treated with salt (NST) and children treated with additional salt at least between two visits between birth and 365 days of life (ST), (n).
