# Supplementary material for: Treatment of congenital adrenal hyperplasia in children aged 0–3 years: a retrospective multicenter analysis of salt supplementation, glucocorticoid and mineralocorticoid medication, growth and blood pressure
Source: Eur J Endocrinol. 2022 Mar 15;186(5):587–96. doi: 10.1530/EJE-21-1085 (PMC9066592; doi:10.1530/EJE-21-1085)
Supplement: Supplementary Table 2: Blood pressure (RR) percentiles from birth to 3 years of age compared by salt replacement status (mean ± SD (n)). [file supplementary_table_2.pdf]

Supplementary Table 2: Blood pressure (RR) percentiles from birth to 3 years of age compared by salt replacement status (mean ± SD (n)).

Age in months		total	NST	ST	p-value*
0	Systolic RR	69 ± 35 (23)	69 ± 40 (5)	69 ± 35 (18)	0.97
	Diastolic RR	89 ± 12 (22)	93 ± 10 (5)	88 ± 13 (17)	0.42
3	Systolic RR	80 ± 28 (59)	89 ± 16 (14)	77 ± 31 (45)	0.17
	Diastolic RR	91 ± 12 (58)	95 ± 5 (14)	90 ± 14 (44)	0.18
6	Systolic RR	81 ± 25 (66)	87 ± 22 (19)	79 ± 26 (47)	0.24
	Diastolic RR	91 ± 18 (64)	94 ± 13 (19)	89 ± 19 (45)	0.27
9	Systolic RR	78 ± 27 (65)	85 ± 21 (17)	75 ± 29 (48)	0.19
	Diastolic RR	92 ± 13 (64)	94 ± 13 (17)	91 ± 12 (47)	0.42
12	Systolic RR	80 ± 28 (71)	86 ± 24 (24)	76 ± 29 (47)	0.15
	Diastolic RR	91 ± 14 (70)	89 ± 16 (24)	91 ± 13 (46)	0.65
18	Systolic RR	83 ± 26 (67)	83 ± 30 (20)	83 ± 24 (47)	0.95
	Diastolic RR	93 ± 13 (67)	94 ± 12 (20)	93 ± 14 (47)	0.76
24	Systolic RR	79 ± 26 (89)	85 ± 21 (28)	76 ± 28 (61)	0.10
	Diastolic RR	89 ± 16 (87)	91 ± 10 (28)	88 ± 18 (59)	0.45
30	Systolic RR	80 ± 22 (82)	86 ± 15 (31)	76 ± 25 (51)	0.05
	Diastolic RR	90 ± 14 (82)	91 ± 14 (31)	88 ± 15 (51)	0.46
36	Systolic RR	70 ± 27 (115)	72 ± 27 (50)	69 ± 28 (65)	0.54
	Diastolic RR	81 ± 21 (114)	80 ± 22 (49)	82 ± 20 (65)	0.63

*p-value: group difference tested between children not treated with salt (NST) and children treated with additional salt at least between two visits between birth and 365 days of life (ST), (n).
